# Advanced polymeric metal/metal oxide bionanocomposite using seaweed *Laurencia dendroidea* extract for antiprotozoal, anticancer, and photocatalytic applications

**DOI:** 10.7717/peerj.15004

**Published:** 2023-03-20

**Authors:** Musarat Amina, Nawal M. Al Musayeib, Seham Alterary, Maha F. El-Tohamy, Samira A. Alhwaiti

**Affiliations:** 1 Department of Pharmacognosy, College of Pharmacy, King Saud University, Riyadh, Saudi Arabia; 2 Department of Chemistry, College of Science, King Saud University, Riyadh, Saudi Arabia

**Keywords:** *Laurencia dendroidea*, Silver nanoparticles, Copper nanoparticles, Polymericbionanocomposite, Antiprotozoal, Anticancer, Photocatalytic

## Abstract

**Background:**

Biosynthesized nanoparticles are gaining popularity due to their distinctive biological applications as well as bioactive secondary metabolites from natural products that contribute in green synthesis.

**Methodology:**

This study reports a facile, ecofriendly, reliable, and cost-effective synthesis of silver nanoparticles (AgNPs), copper oxide nanoparticles (CuONPs), and polymeric PVP-silver-copper oxide nanocomposite using ethanol extract of seaweed *Laurencia dendroidea* and were evaluated for antiprotozoal, anticancer and photocatalytic potential. The nanostructures of the AgNPs, CuONPs, and polymeric PVP-Ag-CuO nanocomposite were confirmed by different spectroscopic and microscopic procedures.

**Results:**

The UV-vis spectrum displayed distinct absorption peaks at 440, 350, and 470 nm for AgNPs, CuONPs, and polymeric Ag-CuO nanocomposite, respectively. The average particles size of the formed AgNPs, CuONPs, and Ag-CuO nanocomposite was 25, 28, and 30 nm, respectively with zeta potential values −31.7 ± 0.6 mV, −17.6 ± 4.2 mV, and −22.9 ± 4.45 mV. The microscopic investigation of biosynthesized nanomaterials revealed a spherical morphological shape with average crystallite sizes of 17.56 nm (AgNPs), 18.21 nm (CuONPs), and 25.46 nm (PVP-Ag-CuO nanocomposite). The antiprotozoal potential of green synthesized nanomaterials was examined against *Leishmania amazonensis* and *Trypanosoma cruzi* parasites. The polymeric PVP-Ag-CuO nanocomposite exerted the highest antiprotozoal effect with IC_50_ values of 17.32 ± 1.5 and 17.48 ± 4.2 µM, in contrast to AgNPs and CuONPs. The anticancer potential of AgNPs, CuONPs, and polymeric PVP-Ag-CuO nanocomposite against HepG2 cancer cell lines revealed that all the nanomaterials were effective and the highest anticancer potential was displayed by PVP-Ag-CuO nanocomposite with IC_50_ values 91.34 µg mL^−1^ at 200 µg mL^−1^ concentration. Additionally, PVP-Ag-CuO nanocomposite showed strong photocatalytic effect.

**Conclusion:**

Overall, this study suggested that the biogenic synthesized nanomaterials AgNPs, CuONPs, and polymeric PVP-Ag-CuO nanocomposite using ethanol extract of seaweed *L. dendroidea* possesses promising antiprotozoal anticancer and photocatalytic effect and could be further exploited for the development of antiprotozoal and anticancer therapeutics agents.

## Introduction

Nanotechnology is primarily concerned with the shape, controlled dispersion of nanoparticles with small size (1–100 nm), chemical composition, synthesis, and their utilization for human benefit (Caputo et al., 2021; Aflori, 2021). Nanotechnology has tremendous potential to create diagnostic solutions, cure, and prevention of ailments at the cellular level, and its application in the medical industry is known as nanomedicine. Nanomedicine encompasses a number of diverse areas, including regenerative medicine, drug delivery systems, and diagnostics and treatment (Mahmoudi, 2021). Metallic nanoparticles play a crucial role in the pharmaceutical and medical sciences.

Among various metal nanoparticles, biogenic silver and copper oxide nanoparticles are synthesized as multifunctional therapeutic materials that provide the advantage of biomedical and pharmaceutical applications with very low systemic toxicity. Silver nanoparticles (AgNPs) have advanced physicochemical properties such as chemical stability, non-linear optical behavior, enhanced thermal and electrical conductivity, high surface Raman scattering, and various biomedical potential (Lee & Jun, 2019). Silver nanoparticles (AgNPs) have been reported to have an enormous number of applications, particularly in biomedicine due to their broad range of biological potential including antibacterial, antiviral, antiprotozoal, antifungal, and anticancer properties (Jain et al., 2021; Sofi et al., 2021). Besides silver nanoparticles, versatile features of copper oxide (CuO) nanoparticles have attained much attention in recent times due to their diverse applications in many scientific fields including heterogeneous catalysts, solar cells, gas sensors, lithium-ion batteries, and antibacterial agents (Grigore et al., 2016). CuO nanoparticles (CuONPs) are the most stable, and robust with a longer shelf-life period in contrast to organic components, and potential antimicrobials (Zheng et al., 2021). Biogenic copper oxide nanoparticles (CuONPs) have been revealed to exhibit various biological properties including antibacterial, antifungal, antioxidant, and anticancer (Amin et al., 2021; Mani et al., 2021). In spite of numerous multifarious applications of monometallic nanoparticles, advancement in the formation of bimetallic hybrid nanoparticles has also been boosted. The combination of metals added an advantage to the combined properties of individual metals. The combined metals contribute to the area of catalysis, reactivity as well as elevated biological properties which offers numerous benefits over their individual metallic counterparts (Sharma et al., 2019).

Despite various conventional approaches for the preparation of nanomaterials, scientists have shifted their focus to the biogenic pathways due to their environmentally benign approach (Aboyewa et al., 2021). The formation of nanostructures by this route offers tremendous advantages including, less-toxic, economical, and biocompatible compared to physical and chemical procedures. The advantages of green or biological synthesis include the ability to readily scale up to large-scale synthesis and the ability to produce nanoparticles in the right sizes and shapes with improved stability (Ahmed et al., 2022; Restrepo & Villa, 2021). Green synthesis reduces the usage of potentially dangerous industrial chemicals while producing nano-products in a single step (Sagandykova, Szumski & Buszewski, 2021). The use of natural resources (plants, diatoms, bacteria, fungi, Yeast, and marine resources) is gaining enormous attention in scientific research because these natural products serve as reducing, capping, and stabilizing agents for the formation of nanoparticles (Kharissova et al., 2019). The type of natural resource extract used to play important role in the size and shape of nanoparticles as a different natural resource has different mounts of reducing content (Makarov et al., 2014). The extracts of natural products are mainly contained abundant bioactive molecules such as phenolics, steroids, terpenoids, tannins, flavonoids, alkaloids, proteins, sugars, and enzymes (Nyamai et al., 2016).

The enormous biodiversity present in marine ecosystems offers a promising resource of novel bioactive compounds with potential human utility (Ameen, AlNadhari & Al-Homaidan, 2021). Some of these microorganisms can survive in harsh marine environments, resulting in complex components with unique biological features that can be utilized in various industrial and biotechnological applications (Menaa et al., 2020). Marine-based bioactive components can be derived from diverse sources, including microorganisms, marine plants, micro and macroalgae, and sponges, all of these contain their own set of a unique set of biomolecules (Malve, 2016). Macroalgae also known as seaweed, represent 23.4% of the tonnage and 9.7% of the value of the global aquaculture (marine, freshwater, and brackish water) production, analyzed at 59.4 million tones and $ 70.3 billion in 2004 (Ghosh, Banerjee & Mitra, 2012). Seaweeds are commonly susceptible to microbial colonization and produce a huge number of secondary metabolites to protect themselves against herbivory and biofouling (Mrid et al., 2021). Seaweed contains various inorganic and organic compounds, including terpenoids, carotenoids, sterols, chlorophylls, xanthophylls, phycobilins, tocopherol, polysaccharides, polyunsaturated fatty acids, vitamins and phycocyanins (Das et al., 2011). These organic constituents serve as phytochemist-attractants, bioprotectant, biostimulant, and microbial nutrient sources as well as mediate in competitive interactions for space in benthic habitats, acting as allelochemicals (Mrid et al., 2021). They are used as food, feed, fodder, and fertilizer, and many bioactive components produced by macroalgae are known to have potential beneficial use in healthcare (Kadam & Prabhasankar, 2010; Smit, 2004). Seaweed species of the genus *Laurencia* have attracted substantial attention from scientists for the untapped diversity of secondary metabolites, particularly terpenes and acerogenins (Cikos et al., 2021). The pharmacological properties of these constituents include potential antibiotic, antiviral, antileishmanial, antimalarial, antitrypanosomal, anti-carcinoma, anticancer, anti-inflammatory, and anti-diabetic activities (Minamida et al., 2021; Cikos et al., 2021). *Laurencia dendroidea* is a red seaweed species widely distributed in the Atlantic Ocean, native to the Brazilian coast. It is found at a 3 m depth from the intertidal to the subtidal zone. An erect violet-greenish or brown-purple-colored thalli forming 4–20 cm dense tufts (Cassano et al., 2012). The macroalgal genus *Laurencia* has been investigated chemically and pharmacologically since 1960, but has become the subject of great interest, as evidenced by the recent discovery of new phytoconstituents, mainly halogenated constituents (Vairappan et al., 2001). Polyphenols, terpenes, and halogenated compounds are the main components of *L. dendroidea* and are reported to possess promising biological properties (Gonçalves et al., 2020; Barcellos et al., 2018). A major halogenated sesquiterpene (-)-elatol produced by *L. dendroidea* exhibited strong biocidal and anti-epibiotic effects (De Oliveira et al., 2015). It can be utilized for the preparation of antifouling paints and the development of antimicrobials (Da Gama et al., 2002). A recent study revealed the (-)-elatol concentration variability in the intra and interpopulation levels in *L. dendroidea*, indicating that this variability could be due to environmental factors such as salinity, and temperature (De Oliveira et al., 2015). Various biosynthetic nanomaterials such as silver, gold, zinc oxide, and Ag-ZnO composite using seaweed *Codium capitatum* P.C., *Fucus gardeneri* (Kannan et al., 2013; Princy & Gopinath, 2021), *Turbinaria conoides* (Rajeshkumar et al., 2013), seaweeds of gulf of Mannar (Nagarajan & Arumugam Kuppusamy, 2013) and *Padina gymnospora* seaweed extract (Rajaboopathi & Thambidurai, 2018) and all these nanoparticles have shown interesting biological properties. There are only few studies reported in the literature for the synthesis of Ag and Au nanoparticles using the marine alga *Laurencia catarinensis* (Abdel-Raouf et al., 2017), red algae *Laurencia aldingensis* and *Laurenciella sp.* (Vieira et al., 2016). To the best of our knowledge, no study has been reported for biogenic synthesis of nanomaterials using *L. dendroidea* till date.

Keeping into consideration the chemical profile and biological properties *L. dendroidea*, the present study focused on the green synthesis of Ag, CuO, and PVP-Ag–CuO NCS using seaweed *L. dendroidea* extract and evaluating their antiprotozoal, anticancer, and photocatalytic activities.

Herein, a facile, environmentally benign, cost-effective, and easy approach was used to prepare Ag, CuO, and polymeric PVP-Ag–CuO nanoparticles using seaweed *L. dendroidea* extract. The as-prepared nanoparticles (AgNPs, CuONPs, and PVP-Ag–CuONPs) were characterized by different spectroscopic including analytical such as ultraviolet–visible (UV-vis), fourier transform infrared (FTIR), and X-ray diffraction (XRD) as well as microscopic such as scanning electron microscope (SEM), Energy dispersive X-ray (ESI), and transmission electron microscope (TEM) methods. In addition, the prepared nanomaterials were evaluated for antiprotozoal activity against *L. amazonensis* and *T. cruzi* parasites. The anticancer potential of nanomaterials was determined against HepG2 cancer cell lines. Also, the photocatalytic effect of biosynthesized was tested towards methylene blue dye.

## Material & Methods

### Chemical and reagents

Methanol (98.2%), ethanol (95.0%), dimethyl sulfoxide (DMSO), silver nitrate (AgNO_3_, ≥ 99%), copper (II) acetate monohydrate (Cu(CO_2_CH_3_)_2_ H_2_O, ≥ 99%), sodium hydroxide (NaOH, 97%), glucose (≥ 99.5%), polyvinylpyrrolidone (PVP), hemin (≥ 90.0%), fetal bovine serum (FBS), ampicillin (96.0%), streptomycin, resazurin, glucantine (≥ 98.0%), benznida-zole (97.0%), 3-(4,5-dimethylthiazol-2-yl)-2, 5-diphenyl tetrazoliumbromide (MTT), tri-buffer, 2-nitrobenzoic acid (DTNB, 95.0%), thiobarbituric acid (TBA, ≥ 98.0%) used for this study were purchased from Sigma-Aldrich (Hamburg, Germany).

### Antiprotozoal strains and cancer cell line

Antiprotozoal parasites, *Leishmania amazonensis* (MNYC/BZ/62/M384), and anti-*Trypanosoma cruzi* (RA) were obtained from the microbiology Department, King Saud University, Saudi Arabia. Human liver cancer cell lines HepG2 (ATCC-HB-8065) were procured from the American Type Culture Collection (ATCC®, Manassas, VA, USA).

### Biomass material

The red macroalga *Laurencia dendroidea* biomass was collected at 1–2 m depth from the coast of Jeddah, Saudi Arabia, in September 2020. The collected biomass was washed with seawater to clean from necrotic and epiphytes parts at the sampling station and shifted to the laboratory under cold conditions. The sample biomass was then rinsed with sterile water, followed by ethanol (75%) to it free from contaminating materials and associated microflora. The collection of microalgae was supported by identification authorization LD 33241–1(Microbiology department, KSU, Riyadh, Saudi Arabia).

### Preparation of biomass extract

The dried powder of macroalga *L. dendroidea* (100 g) was subjected to Soxhlet extraction using 95% of ethanol (3 × 500 mL). All the collected extracts were combined, filtered through Whatman filter paper No 1, and concentrated at 50 °C under reduced pressure on a rotavapor. A green-colored residue (12.3 g) was obtained and stored in the refrigerator until further use.

### Preparation of Ag, CuO, and polymeric PVP-Ag–CuO NCS

**Scheme 1:**
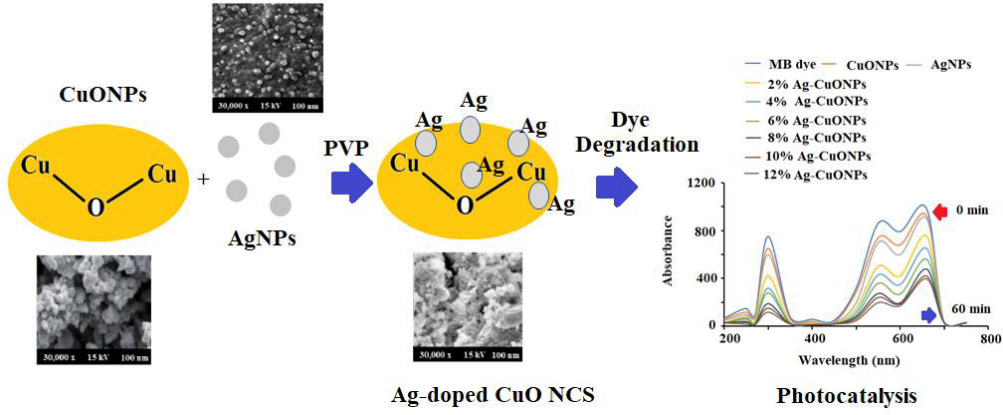
Schematic synthetic mechanism of formation of polymeric Ag-CuO NCS.

The *L. dendroidea ethanolic* extract (2 g) was dissolved in 100 mL Millipore water and stirred for 2 h under sonication. The 10 mL of dissolved material was treated with 0.5 g silver nitrate under continuous stirring for ∼2 h at 80−85 °C. The instant color change from greenish-yellow to dark brown of the reaction mixture occurred, afterward, no color transformation was observed till the reaction ended. The reaction mixture was then cooled down and centrifuged at 10,000 for 30 min. Consequently, the resulting product was washed thoroughly several times using Millipore water. Finally, the obtained black precipitate was collected and dried at 80 °C for 12 h in a domestic oven. The reduced Ag^+^ ions were spectrophotometrically monitored between 190–800 nm. Whereas the CuONPs was prepared by dissolving 5.0 g of Cu (CO_2_CH_3_)_2_ H_2_O in 10 mL of distilled water under constant magnetic stirring at ambient temperature for 10 min. Afterward, 2.0 g of *L. dendroidea* extract dissolving in 10 mL deionized water was added dropwise to the copper acetate solution under constant stirring at 60 °C for 4 h. A blackish precipitate appeared after the complete addition of *L. dendroidea* extract which was heated to evaporate the excess water for a further 30 min. After the complete removal of water, the sample calcined at 400 °C for 4 h in a furnace oven resulting in the formation of CuONPs. The pre-calcined CuONPs were cooled at ambient temperature prior to exploration. However, PVP-Ag–CuO NCS was prepared with copper acetate, silver nitrate, and *L. dendroidea* extract in the presence of a polymeric matrix. Briefly, 0.2 mol L^−1^ of copper acetate was dissolved in 10 mL of 1.0% PVP (w/v) dissolved in deionized water and 0.42 g of silver nitrate (0.025 M) was added slowly to the solution under magnetic stirring for 15 min at ambient temperature. 2 g of *L. dendroidea* extract dissolved in 10 mL deionized water was added to the above reaction mixture with constant stirring for 20 min and instant color change to dark black suggested the formation of PVP-Ag–CuO NCS. PVP serve as a surface stabilizer, nanoparticle dispersant, reducing agent and growth modifier in the formation of nanomaterial. Afterward, the solution mixture was heated for 15 min at 80 °C and the final resulting product was washed three times with ethanol followed by deionized water. The formed PVP-Ag–CuO NCS was allowed to dry in vacuum desecrator (Scheme 1). Previous studies have shown that nanoparticles such as AgNPs, CuONPs and bionanocomposite were also prepared by using plant extract, sponges, bacteria, proteins, and polysaccharides. A tabulated comparison of biosynthetic method, source used and their application were illustrated in Table 1.

### Characterization

UV–Visible DRS (UV-2600 Shimadzu, Kyoto, Japan) was applied to determine the surface plasmon resonance (SPR) and optical properties of AgNPs, CuONPs, and polymeric PVP-Ag–CuO NCS. The structural information and crystalline phase of pre-synthesized nanoparticles and nanocomposite were obtained with Cu K*α* (1.5405 Å) in 40 mA and 30 kV using a ProPowder X’Celerator diffractometer PANalytical X’Pert (Malvern, UK). The functional moieties and bonds in pre-synthesized nanomaterials were verified by FT-IR in 4000–400 cm^−1^ on Perkin Elmer (Llantrisant, United Kingdom). SEM (CarlZeiss, Oberkochen, Germany), TEM (Titan, Corston Bath, United Kingdom), and EDX (ThermoFisher Scientific, Waltham, MA, USA) were applied to measure the surface morphology, nano-sizes, and elemental composition of prepared nanoparticles and nanocomposite.

### Antiprotozoal activity

The antiprotozoal activity of AgNPs, CuONPs, and PVP-Ag–CuO NCS was evaluated by anti-*Leishmania* and anti-*Trypanosoma* activity assays by obeying the described methods of (Aguilera et al., 2019; Aguilera et al., 2019). *L. amazonensis*, promastigotes were cultured in an axenic medium containing BHI-Tryptose provided with 3.0 × 10^−4^ g/mL glucose, 2 × 10^−5^ mg mL^−1^ hemin, 10% of fetal bovine serum (FBS), 1.3 × 10^−4^ g mL^−1^ ampicillin, and 2.0 × 10^−4^ g mL^−1^ streptomycin at 28 °C (Faral-Tello et al., 2020). 96-well plates were loaded with 2 × 10^6^ promastigotes/well for the assay. The test samples (AgNPs, CuONPs, and PVP-Ag–CuO NCS) were dissolved in dimethyl sulfoxide (DMSO). 200 µL of each sample of different concentrations (25−0.05 µg mL^−1^) were added separately to individual well-plate and incubated at 28 °C for 48 h. Afterward, 20 µL of a resazurin solution (2 µg mL^−1^ prepared in PBS, pH 7.4) was added to each well plate. The quantification of oxidation–reduction of the reaction mixture was noted at 570 and 600 nm. The efficacy of pre-synthesized nanoparticles and nanocomposite was measured by determining the IC50 values using OriginLab 8.5® sigmoidal regressions. Glucantime was used as the reference standard. Each applied concentration was examined in triplicates and each anti-proliferative study was performed in duplicate. However, epimastigotes were cultured in an axenic medium (BHI-Tryptose), for the anti-*T. cruzi* potential (*in vitro*). 5–7 days old cell cultures were loaded in a freshly prepared culture medium to obtain 2 × 10^6^ cells/mL initial concentration. Each day absorbance of the cell culture was recorded at 600 nm. On the 5th day, different doses (25−0.05 µg mL^−1^) of the test sample dissolved in 0.4% (v/v) DMSO were inoculated onto the medium from the stock solution. Medium containing 0.4% DMSO v/v was used to cultivate the control parasites and benznidazole was applied as a positive control. The measured absorbance on the 5th day was compared with the control and IC50 values were estimated for each test sample using OriginLab 8.5® sigmoidal regressions. Each applied concentration was examined in triplicates and each anti-proliferative study was performed in duplicate.

**Table 1:** Comparison of biosynthetic method with the extract of other natural sources for the preparation of AgNPs CuONPs and bionanocomposite.

Biogenic Nanoparticles	Natural product used	Source	Shape of nanoparticles	Particle size	Reference
AgNPs	*Acer oblongifolium* fresh leaves	Plant extract	Rod-like	5–8 nm	Naveed et al. (2022)
	*macroalga Chaetomorpha linum*	Marine Algae	Cubic structure	30–35 nm	Kannan et al. (2013)
	*Lysiloma acapulcensis*	*Bacteria*	quasi-spherical	5 nm	Garibo et al. (2020)
	Marine macro algae	Polysaccharides	Spherical	20 nm	El-Rafie, El-Rafie & Zahran (2013)
CuONPs	*Malva sylvestris* leaf extract	Plant extract	Spherical	19–28 nm	Benhammada & Trache (2022)
	Sugarcane extract	Polysaccharides	–	29–55	Alturki (2022)

### Anticancer activity

The anticancer activity of *L. dendroidea* extract, AgNPs, CuONPs, and PVP-Ag–CuO NCS was examined on the HepG2 cancer cell lines using cell viability and MTT assays. The effect of pre-synthesized nanomaterials on the viability of HepG2 cancerous cells was carried out by obeying the MTT procedure. In 24-well microtiter plates the HepG2 cells with (1.0 × 10^4^ cells/well) density were loaded in a 1.0 mL culture medium. About 50–200 µg mL^−1^ dilution of pre-synthesized nanomaterials was added to each microtiter plate and incubated at 37 °C for 48 h in a humidified incubator. Afterward, 200 µL of MTT reagent prepared in phosphate-buffered saline (PBS, 5 mg mL^−1^, pH 7.4) was added to each well plate and placed undisturbed for 2 h at room temperature. Finally, the reaction mixture solution was treated with 200 µL of DMSO and subjected to spun (1800 × g) at 4 °C for 5 min. The absorbance was noted at 540 nm on an Elx-800 microplate reader (Eid & Hawash, 2021). The effect of AgNPs, CuONPs, and polymeric PVP-Ag–CuO NCS on the growth inhibition was calculated as percentage cell viability in contrast with DMSO-treated cells as control. The values of test samples were used to subtract the absorbance number of media-containing wells.

\begin{eqnarray*}\text{% Cell Viability}=({A}_{\mathrm{s}}-{A}_{\mathrm{b}})/({A}_{\mathrm{c}}-{A}_{\mathrm{b}})\times 100 \end{eqnarray*}

Where A_s_, A_b_, and A_c_ represent the absorbance’s of test samples, blank, and control, respectively.

### Estimation of protein (Bradford assay)

The protein concentration was evaluated using the Bradford assay in the cell pellets obtained from AgNPs, CuONPs, and polymeric PVP-Ag–CuO NCS-treated HepG2 cancer cells (Afsar et al., 2016).

### GSH levels determination in HepG2 cancer cell line

The nanomaterials-treated HepG2 cancerous cells were centrifuged at 9,000 rpm for 10 min until the cellular protein was precipitated out. Subsequently, 0.4 mol L^−1^ of tris buffer (pH 8.9) was added to the supernatant, followed by the addition of DTNB reagent, and incubated at for 10 min under constant shaking. The change of color was noticed and the intensity of color was recorded at 412 nm (Okuno et al., 2003).

### Lipid peroxidation estimation in HepG2 cancer cells

Lipid peroxidation (LPO) quantitation was performed using TBA reactive substances (TBARS) assay using a TBARS kit. Nanomaterials-treated HepG2 cancerous cells were centrifuged at 9,000 rpm for 10 min, sonicated to make a uniform solution, and centrifuged again under similar conditions. After centrifugation, the supernatant was collected, and about 500 µL of supernatant was reacted with one mL of TBA and incubated at 100 °C for 15 min in a water bath. The reaction mixture solution was cooled and then centrifuged for 2 min at 13,000 × g. A lysate supernatant (500 µL) was separated and at 550 nm absorbance of the fluorescent adduct was recorded. TBARS were represented as minimum detectable activity (MDA) equivalents (Rao et al., 2019).

### Photocatalytic effect

The photocatalytic effect of AgNPs, CuONPs, and polymeric PVP-Ag–CuO NCS was evaluated by the degradation of methylene Blue (MB) as a reference pollutant. A Xe lamp with 300W in combination with a UV cutoff filter was applied as a source of light to obtain visible light of *λ*_max_ ≥ 400 nm wavelength, and illumination intensity of 80 mW cm^−2^. A 0.031 mM solution of MB dye was prepared by dissolving 0.01 g of MB dye in 100 mL of deionized water and stored under a dark condition at room temperature as a stock solution. Afterwards, each test sample (0.1 g) was dispersed in separate customized reactor in 100 mL of 0.031 mM of MB solution. Each obtained sample suspension solution was kept in the dark under a constant magnetic stirring for 30 min to achieve an adsorption–desorption equilibrium between photocatalyst and MB as well as good dispersion. The sample suspensions were exposed to sunlight. After every 5 min interval in the course of visible light exposure, around 10 mL of the reaction mixture was taken out and subjected to centrifugation to eliminate the trace solid particles. Finally, the visible absorption spectra were measured to study the photocatalytic effect of each test sample.

## Results & Discussion

### Characterization of synthesized nanoparticles

**Figure 1:**
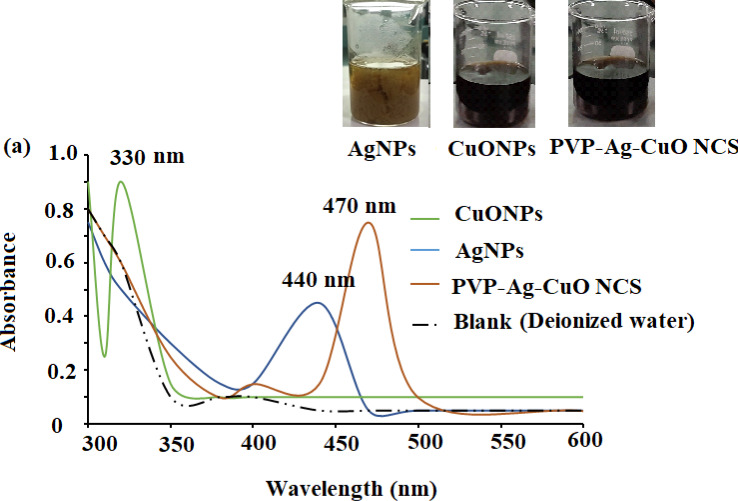
UV-Vis spectra of CuONPs (350 nm), AgNPs (440 nm), and polymeric PVP-Ag/CuO nanocomposite (470 nm).

**Figure 2:**
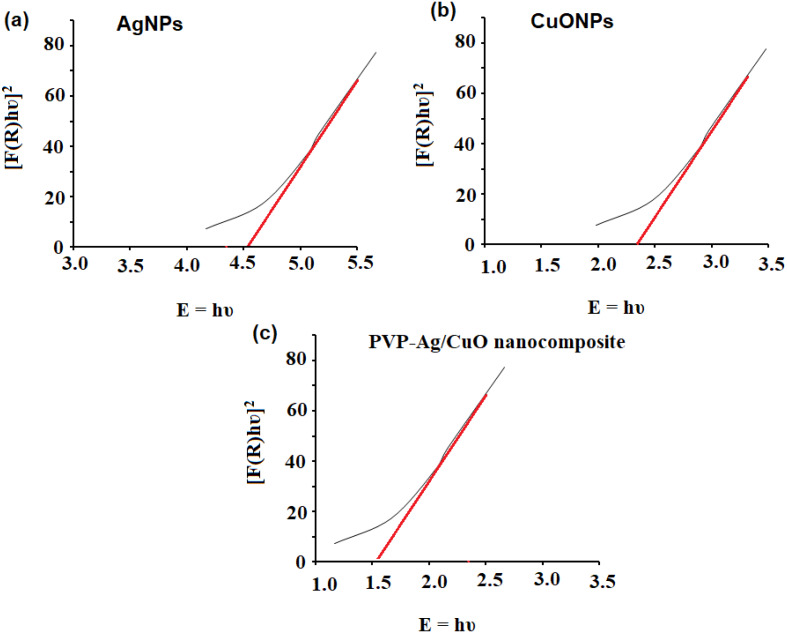
Bandgap energy of (A) AgNPs, (B) CuONPs, and (C) PVP-Ag/CuO nanocomposite.

The formation of biogenic synthesized AgNPs, CuONPs, and polymeric PVP-Ag–CuO NCS was confirmed using UV-Vis spectrophotometric measurement at absorption wavelength in the range of 200–600 nm. The colloidal suspensions of the pre-synthesized nanomaterials displayed three absorption maxima at 440, 330, and 470 nm, for AgNPs, CuONPs, and polymeric PVP-Ag–CuO NCS, respectively (Fig. 1). The optical characteristic of these nanomaterials in the visible region was due to the absorption from the collective oscillation of electrons as a result of the electric field of electromagnetic radiation light (Kumar et al., 2012). Tauc’s plot was used to calculate the bandgap using Eg = h υ = hc/*λ* equation, where h, c, and *λ* represent Planck’s constant, the velocity of light, and the wavelength, respectively. The estimated bandgaps were found to be 4.51 eV, 2.40 eV, and 1.56 eV, for AgNPs, CuONPs, and PVP-Ag–CuO NCS, respectively (Figs. 2A–2C). The difference in bandgap energy between AgNPs and polymeric PVP-Ag–CuO NCS was 2.95 eV, while the bandgap energy difference between CuONPs and polymeric PVP-Ag–CuO NCS was 0.84 eV, attributed to redshift. The decrease in bandgap energy in polymeric PVP-Ag–CuO NCS enhances the electrons active in oxidation. Moreover, the surface plasma resonance increases the radiation penetration ability, generates the scattering probability, and provides the reduction form to the surface sites to the whole movement on the surface of CuO surface, and their interaction may elevate the process. These processes involve the whole generation and separation of electrons on the surface, improving the process of oxidation.

**Figure 3:**
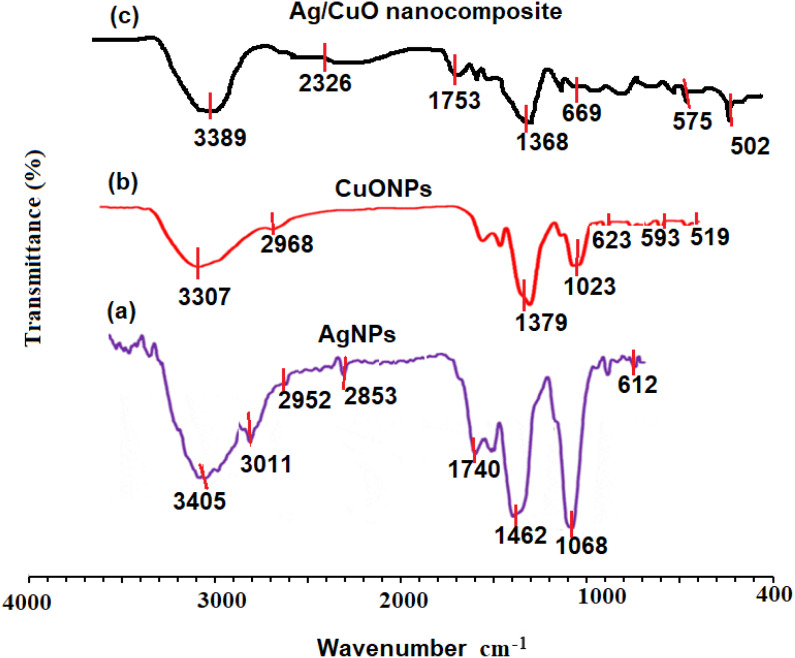
FT-IR spectra of (A) AgNPs, (B) CuONPs, (C) polymeric PVP-Ag/CuO nanocomposite, and (d) seaweed extract measured at 4000–400 cm^−1^.

The FT-IR spectra of pre-synthesized AgNPs, CuONPs, and polymeric PVP-Ag–CuO NCS were recorded. The FT-IR of AgNPs showed significant absorption bands at 3405, 3011, 2952, 2853, 1740, 1462, and 1068 cm^−1^. The absorption bands at 3405 and 3011 cm^−1^ can be assigned for O-H and N-H stretching vibration which indicates the presence of some polyphenolic constituents in the *L. dendroidea* extract attached to the surface of AgNPs. The bands appeared at 2952 and 2853 cm^−1^ indicated the presence of C-H alkane and aldehyde stretching vibration groups, respectively. The three bands observed at 1740, 1462, and 1068 cm^−1^ can be assigned to C =O, a strong stretching ester group, medium C-H bending methylene group, and strong C-O stretching primary alcohol group, respectively (Fig. 3B). The presence of amine, carbonyl, and alcoholic groups revealed the capping effect of the phytochemicals present in *L. dendroidea* extract on the synthesized AgNPs (Nguyen et al., 2019). However, the FT-IR spectra of CuONPs and polymeric PVP-Ag–CuO NCS were studied and compared. On investigating the spectra of CuONPs and polymeric PVP-Ag–CuO NCS, it was noticed that CuONPs exhibited strong and sharp absorption bands at 400 to 700 cm^−1^. These peaks correspond to undoped Cu-O vibrational mode with significant bands at 519, 593, and 623 cm^−1^ (Rashad et al., 2015; Sharma et al., 2015). Furthermore, it was noticed that the spectrum of CuONPs showed two broad bands at 3307 and 1379 cm^−1^ corresponding to O-H (intermolecular stretching alcohol group) and (C-H bending of dimethyl group), respectively. Whereas, the FT-IR spectrum of polymeric nanocomposite displayed less intense and shifted bands between 500 and 700 cm^−1^ representing the Cu-O with major bands located at 502, 575, and 669 cm^−1^. Also, two broad bands at 2326 and 1753 cm^−1^ were observed which correspond to C =O and *O* = *C* = *O* stretching vibration of anhydride and carbon dioxide, respectively. A similar band noticed at 2326 cm^−1^ in the CuONPs spectrum was less in its intensity indicating the difference in reduction and capping process in the CuONPs and polymeric PVP-Ag–CuO NCS samples (Figs. 3C and 3D). However, the FT-IR spectrum of seaweed extract displayed different absorption bands at 3410, 2921, 2848, 1620, 1382, 1095, 986, and 610 cm^−1^ corresponding to strong O-H stretching of alcohol, weak O-H starching of alcohol, C-H stretching of alkane, C =C stretching of cyclic alkene, medium C-N stretching of amine, strong C =C starching of monosubstituted alkene, strong halo compound stretching vibration, respectively (Fig. 3A). The spectrum of seaweed extracts clearly showed the absence of Ag and CuO peaks in the extract. Thus, the main responsible components for the reduction, capping and stability of AgNPs, CuONPs, and Ag–CuO NCS were sesquiterpenes, phenolics, acids, proteins, and polysaccharides present in the extract of *L. dendroidea* seaweed (Al-Massarani, 2014).

**Figure 4:**
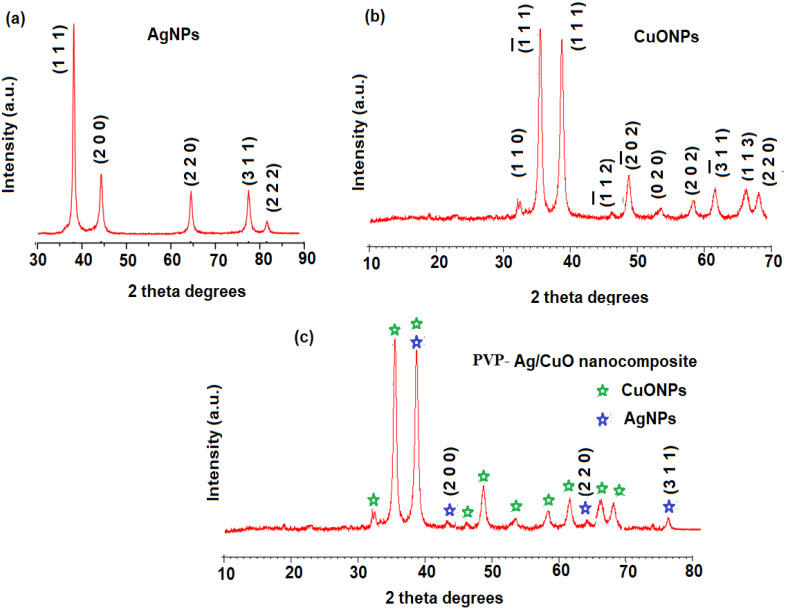
XRD patterns of (A) AgNPs, (B) CuONPs, and (C) polymeric PVP-Ag/CuO nanocomposite.

The XRD estimation of AgNPs, CuONPs, and polymeric PVP-Ag–CuO NCS was established by applying Cu K*α* radiation whose peaks were compared with the Rietveld profile fitting procedure. The XRD pattern of *L. dendroidea* extract-mediated AgNPs showed a similar peak profile to typical obtained for reported AgNPs suggesting a high purity of the pre-synthesized nanoparticles. The 2*θ* values at 38.2° (1 1 1), 46.3° (2 0 0), 64.6° (2 2 0), 77.6° (3 1 1), and 80.4° (2 2 2) were recorded which is indicative of a face-centered cubic crystalline nature of AgNPs (Fig. 4A) and consistent with the database (JCPDS No. 04-0783) (Lanje, Sharma & Pode, 2010). While as, diffraction peaks 2 *θ* values at 32.5° (1 1 1), 35.7° (2 0 0), 46.7° (2 0 2), and 66.7° (1 1 3) were recorded for the plane orientation of monoclinic CuO structure (Fig. 4B) and matched with standard database (JCPDS80-1268) (Skawky & El-Tohamy, 2021). The purity of pre-synthesized CuONPs was confirmed by the absence of secondary Cu_2_O or Cu_4_O_3_ phases. However, the analysis of polymeric PVP-Ag–CuO NCS exhibited four distinctive peaks for the AgNPs at 2 *θ* values of 37.89° (1 1 1), 44.04° (2 0 0), 64.21° (2 0 2), and 76.78° (3 1 1) indicating a crystalline cubic structure, in agreement with the findings of other reported studies (Elango et al., 2018). Thus, the crystalline nature and the production of AgNPs as a result of the green biosynthesis technique were confirmed. As a significant peak for Ag was observed in polymeric PVP-Ag–CuO NCS, it was illustrated that Cu sites were interstitially substituted by Ag without creating any other defects (Fig. 4C). Moreover, the Scherer equation was used to calculate the average size of AgNPs, CuONPs, and polymeric PVP-Ag–CuO NCS by obeying *D* = 0.9 *λ*/*β*Cos*θ* where D, *λ*, *β*, and *θ* represent crystallite size, wavelength, and the half-width of diffraction peak, and the diffraction angle of the highest peak, respectively (Khan et al., 2020). The average crystallite size obtained for AgNPs, CuONPs, and polymeric PVP-Ag–CuO NCS was found to be 17.56 nm, 18.21 nm, and 25. 46 nm, respectively.

**Figure 5:**
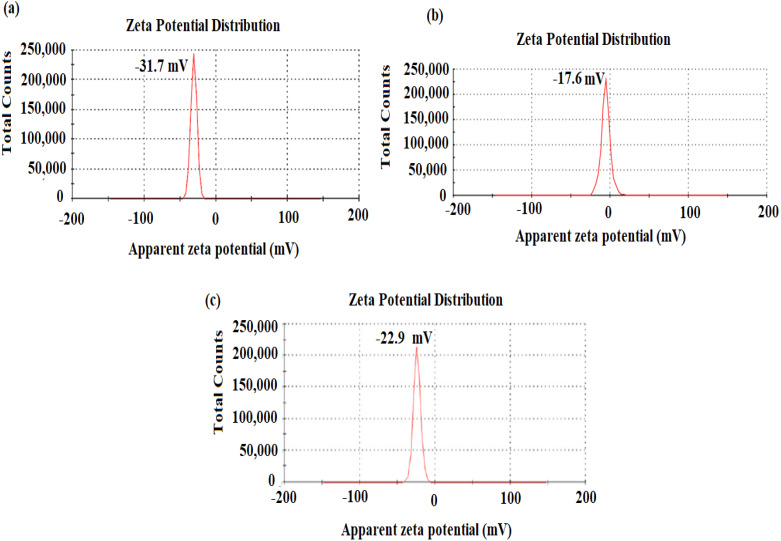
Zeta potential of (A) AgNPs, (B) CuONPs, and (C) polymeric PVP-Ag/CuO nanocomposite.

The stability of nanostructures is crucial for various applications and can be measured by zeta potential (Lunardi et al., 2021). The nanostructures stability is the liquid surface charge of nanomaterials in the solution and is assumed stable when the values of zeta potential are higher than 30 mV or less than −30 mV (Narain, 2020). The surface charge of nano-substances was evaluated using deionized water as dispersant (cP: 0.8872, RI: 1.330, and *ɛ*: 78.5). The net charge of AgNPs, CuONPs, and polymeric PVP-Ag–CuO NCS was observed negative, indicating that the nanostructures were attained by capping with organic constituent present in *L. dendroidea* extract (Figs. 5C–5C). However, zeta potential values obtained for AgNPs, CuONPs, and polymeric PVP-Ag–CuO NCS were −31.7 ± 0.6 mV, −17.6 ± 4.2 mV, and −22.9 ± 4.45 mV, respectively, suggesting the physical stability of obtained nanostructures by the reduction with *L. dendroidea* extract. While the CuONPs reached the delicate dispersion threshold, the polymeric PVP-Ag–CuO NCS, besides the fact, they are considered a similar category, were closer to displaying medium stability levels. Thus, the stability of polymeric PVP-Ag–CuO NCS was found to be enhanced with AgNPs by improving the physical state of CuONPs in the solution.

**Figure 6:**
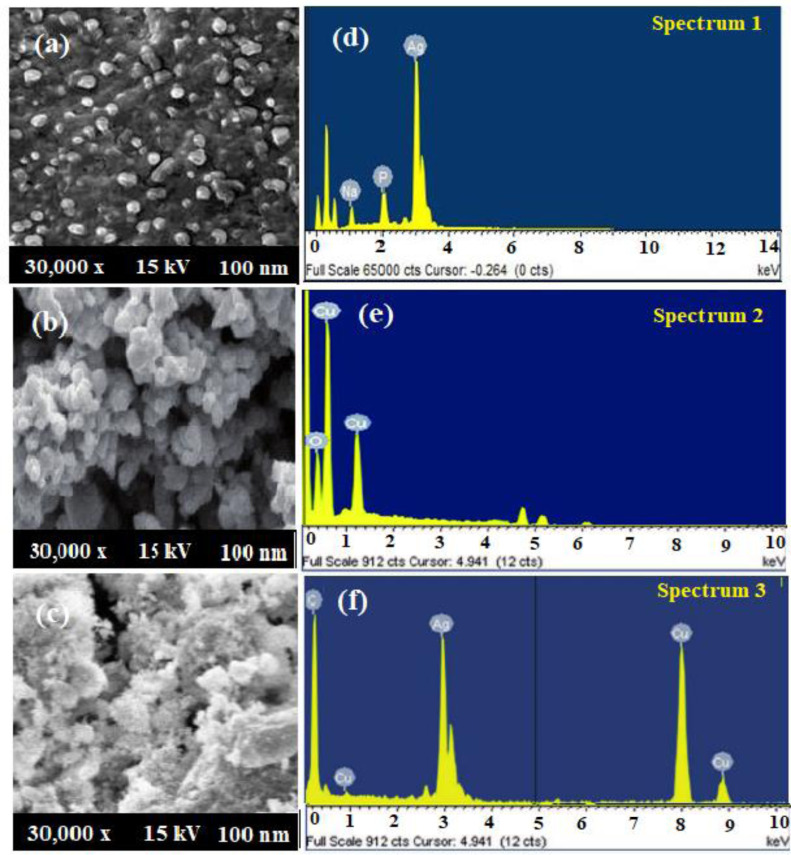
(A–C) SEM spectra and (D–F) EDX AgNPs, CuONPs, and polymeric PVP-Ag/CuO nanocomposite.

The size, surface morphology, and elemental presence in the biosynthesized AgNPs, CuONPs, and polymeric PVP-Ag–CuO NCS were visualized by SEM coupled with EDX. Figure 6A depicted the size of AgNPs in the 19–30 nm range with a 25 nm average size. The images of SEM showed that most of the biosynthesized AgNPs were spherical in shape. Whereas, the SEM images of the pre-synthesized CuONPs revealed a quasi-spherical shape in a 10–40 nm particle size range with a 28 nm average size (Fig. 6B). However, in biosynthesized polymeric PVP-Ag–CuO NCS, surface of CuO was clustered by AgNPs, size, and shape were changed to the lattice arrangement of the nanocomposite. Thus, polymeric PVP-Ag–CuO NCS were found spherical in shape with 20–35 nm range and average size 30 nm (Fig. 6C). The orientation of Ag^+^ ions with the surface of copper oxide (Cu^2+^ and O^2−^), the modification in the size of polymeric PVP-Ag–CuO NCS took place *via* lattice oxygen vacancy occupied by Ag^+^ and Cu^2+^ ions onto the surface (Rehman et al., 2021). The elemental composition of AgNPs, CuONPs, and polymeric PVP-Ag–CuO NCS measured by EDX was shown in Figs. 6D, 6E and 6F. Figure 6D showed the presence of Ag with percentage weights of 98.89% and atomic percentage 92.79%. Figure 6E, displayed the presence of Cu and O with percentages weight 73.67% and 26.33%, atomic percentage 41.33% and 58.67%, respectively. However, the polymeric PVP-Ag–CuO NCS spectrum showed the presence of Ag, Cu, and O elements with weight percentages of 4.54%, 68.23%, and 27.25%, atomic percentages 1.27%, 39.70%, and 59.32% (Fig. 6F), respectively. The low Ag content was reassured by the values obtained in the EDX spectrum.

**Figure 7:**
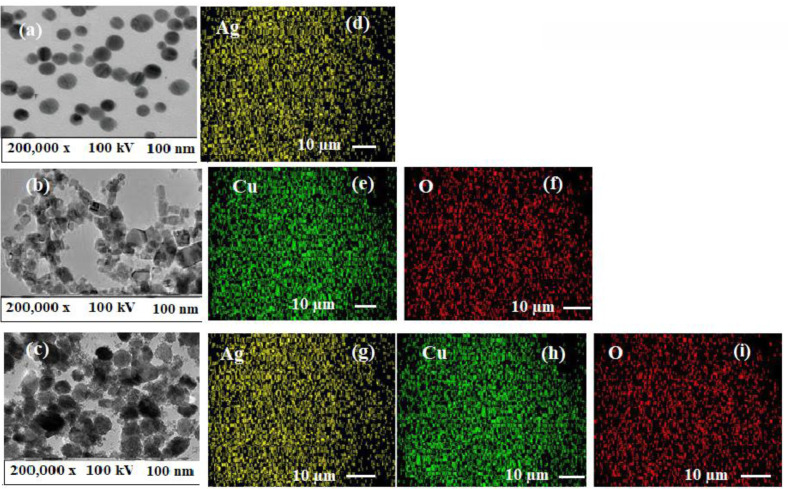
(A–C) TEM images and (D–I) elemental mapping of AgNPs, CuONPs, and polymeric PVP-Ag/CuO nanocomposite.

The spherical shape of AgNPs and polymeric PVP-Ag–CuO NCS as well as the quasi-spherical shape of CuONPs has been visualized in TEM images (Figs. 7A–7C). The average particles size for AgNPs, CuONPs, and polymeric PVP-Ag–CuO NCS were observed at 25 nm, 30 nm, and 32 nm, respectively, which were in agreement with the SEM and XRD analysis. In this study, *L. dendroidea* extract was responsible for the production and growth of Ag/CuO NCS. The potential of Ag^+^ ions is strongly involved than Cu^2+^ ions, whereas the surface morphology of Ag/CuO NCS depicted the formation of Ag cluster onto CuO surface. The measurements of lattice fringes showed 0.23 nm of (1 1 1) and 0.26 nm of (2 0 0) plane, for Ag and CuO, respectively. The decoration of Ag phase on the surface of CuO was due to their atomic orientation relationship and the lattice fringes values were found almost similar in Ag and CuO (Fig. 7C). The components of plant extract were reduced and may be combined with O ions of Cu_2_O nucleate. The Ag cluster was oriented towards CuO surface (Peng et al., 2012). It explores the cubic form that was modified strongly to spherical shape and the polycrystalline nature of pre-synthesized polymeric PVP-Ag–CuO NCS was confirmed by the SAED pattern. The atomic arrangement of biosynthesized AgNPs, CuONPs, and polymeric PVP-Ag–CuO NCS was evaluated by EDX mapping analysis (Figs. 7D–7F). Figure 7D showed the mapping of AgNPs, where Ag ions are spread over the O, while the mapping images of CuONPs showed mutual spreading of copper and oxygen (Figs. 7E and 7F). However, Ag/CuO NCS mapping spectrum exhibited the content of Cu was higher than Ag and O (Figs. 7G, 7H and 7I). Furthermore, the decoration of Ag with Cu and O atoms were noticed in the mapping analysis of polymeric PVP-Ag–CuO NCS.

### Antiprotozoal potential

**Figure 8:**
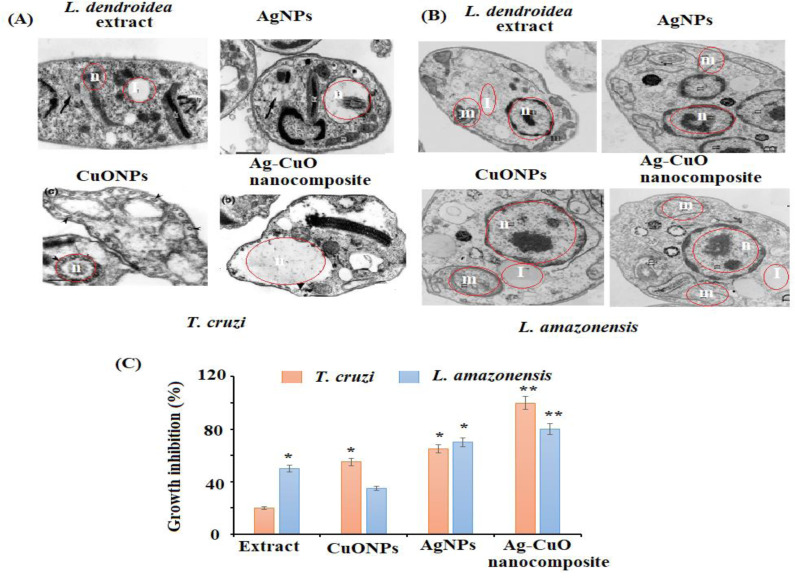
Morphological changes in (A) *T. cruzi*, (B) *L. amazonesis* after treatment of *L. dendroidea*, AgNPs, CuONPs, and polymeric PVP-Ag/CuO nanocomposite, (C) effect of polymeric AgNPs, CuONPs, and polymeric PVP-Ag/CuO nanocomposite (25 µM).

The *in vitro* antiprotozoal efficacy of AgNPs, CuONPs, and polymeric PVP-Ag–CuO NCS were tested against *L. amazonensis* promastigotes and *T. cruzi* epimastigotes (Fig. 8). The polymeric PVP-Ag–CuO NCS was found to be the most active followed by CuONPs towards *T. cruzi* epimastigotes with an IC_50_ value of 17.32 ± 1.0 and 12.36 ± 1.7 µg mL^−1^, respectively at 4.2 µgmL^−1^ concentration. Whereas, AgNPs expressed moderate effect towards *T. cruzi* epimastigotes with IC50 value greater than 25 µg mL^−1^. Benznidazole has been used as positive control with IC50 of 7.28 ± 0.9 µg mL^−1^. However, polymeric PVP-Ag–CuO NCS and AgNPs were most active on *L. amazonensis* promastigotes with IC50 values of 17.48 ± 0.9 (3.9 µg mL^−1^) and 18.75 ± 0.1 (8.0 µg mL^−1^), respectively. The CuONPs were found less active with IC50 >25. The enhanced antiprotozoal properties of the bionanocomposite can be attributed to the combined effect of constituents of *L. dendroidea* extract, AgNPs, CuONPs, and PVP medium. These active bionanocomposite could find application in the area of food agriculture to attain safe and high-quality products (Alarfaj et al., 2021). The amplified effect of bionanocomposite compared to AgNPs and CuONPs toward the tested parasites indicated that the bionanocomposite possesses a combined effect of AgNPs, CuONPs in combination with phytoconstituents present in the extract of *L. dendroidea*. The incorporation of Ag–CuO bionanocomposite in the PVP polymeric medium with active phytoconstituents of *L. dendroidea* has alleviated the antiprotozoal effect of the bionanocomposite. The surface of bionanocomposite decorated with Ag and CuONPs can easily enter the target organelles through the *L. amazonensis*, and *T. cruzi*, leading to disruption of membranes and release of cytoplasm as well as contents of the cell. Subsequently, results in the destruction of parasites. The antiprotozoal activity of the nanoparticles as well as bionanocomposite depends upon various factors such as absorption rate, metabolite release, metabolic processes, and dispersion in the cell (Diez-Pascual & Diez-Vicente, 2014). The nanostructure binds to the microorganism cell, adheres to the surface *via* active. If nanomaterial attaches to or interacts with proteins, a crucial biomolecule in the cell that serves a variety of functions, including the formation of cell components (wall, membrane, nucleic acids, and ribosome) or inhibit protein synthesis inside the cell will stop all protein-related functions, which will lead to cell death (Seillier et al., 2012).

The nanostructure binds with the cell wall of a microorganism, enters into the cell membrane and then disrupts and destroys the membrane (proteins, DNA, and enzymes) (Das et al., 2011). The nanoparticles and nanocomposite generate ROS (hydroxyl radicals OH, hydrogen peroxide H_2_O_2_, and superoxide ions O_2_) inside the cell that causes oxidative stress, which disrupts metabolic processes and causes cell death (He et al., 2011). These free radicals also interact with the biomolecules, disintegrating plasma membrane and leading to lipid oxidation. The nanoparticles and bionanocomposite showed potential antiprotozoal activity in contrast to nanomaterials synthesized chemically. Hence, the adoption of a green approach to prepare nanoparticles and their composites is more plausible for biomedical applications. Further studies and needed to confirm this hypothesis and to establish such targets.

### Anticancer activity

#### Growth inhibition and cell viability of HepG2 cancer cells

**Figure 9:**
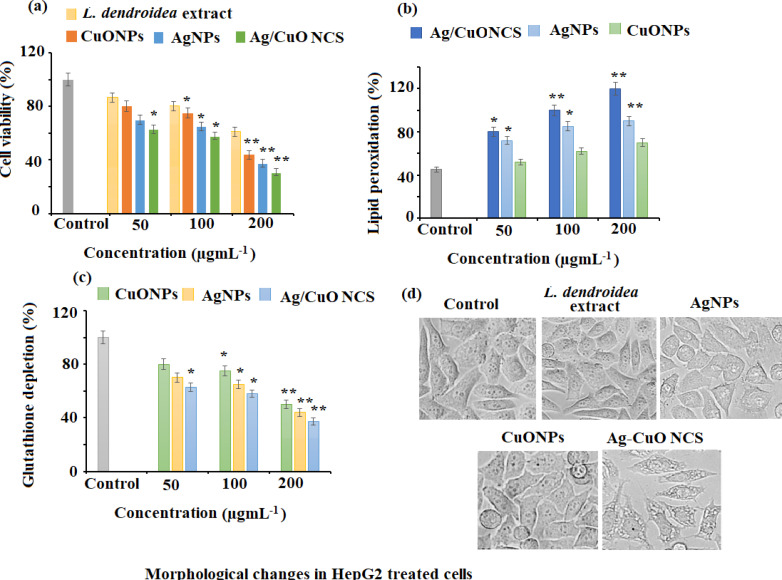
Anticancer effects against HepG2 cell lines. (A) Cytotoxicity assessment by MTT assay. (B) Effect on lipid peroxidation. (C) Percent change in glutathione levels and (D) morphological changes in pretreated HepG2 cells with AgNPs, CuONPs, and polymeric PVP.

The anticancer potential of *L. dendroidea* extract, AgNPs, CuONPs, and polymeric PVP-Ag–CuO NCS was examined by MTT assay against HepG2 cancer cells. The antiproliferative potential of *L. dendroidea* extract, AgNPs, CuONPs, and Ag–CuO NCS was evaluated by MTT assay towards HepG2 cancerous cells. It was noticed that admiration of *L. dendroidea* extract, AgNPs, CuONPs, and polymeric PVP-Ag–CuO NCS at varying concentrations (50–200 µg mL^−1^ for 24 and 48 h) to HepG2 cancer cells resulted in cell growth inhibition in a time and concentration-dependent manner. The results revealed that HepG2 cells respond to *L. dendroidea* extract, AgNPs, CuONPs, and polymeric PVP-Ag–CuO NCS treatment in 48 h, according to time course analysis. However, the polymeric PVP-Ag–CuO NCS showed strong growth inhibition of HepG2 cells followed by AgNPs and CuONPs. The IC50 values of polymeric PVP-Ag–CuO NCS, AgNPs, and CuONPs treated HepG2 were 38.12 ± 0.13, 41.25 ± 0.25, and 52.14 ± 0.25 µg mL^−1^ at (200 µg mL^−1^). However, the *L. dendroidea* extract showed less effect with IC50 value 58.29 ± 0.58 µg mL^−1^ at the same concentration. The obtained data revealed that all the prepared nanomaterials showed significant potential for inhibiting HepG2 cell proliferation. However, polymeric Ag–CuO NCS displayed strong inhibition effects in contrast to AgNPs and CuONPs (Fig. 9A). The changes in the cell cycle regulation in tumor patterns can cause cellular proliferation. The rapid proliferation that resulted in the series and extension of tissue accumulation can be used to identify a significant basic origin of cancer succession (Ayyildiz et al., 2021; Kubczak, Szustka & Rogalinska, 2021). The MTT assay results indicated that AgNPs, CuONPs, and polymeric PVP-Ag–CuO NCS were effective against HepG2 cancer cells. The growth of cells in HepG2 cells was transformed by AgNPs, CuONPs, and polymeric PVP-Ag–CuO NCS. While the administration of polymeric PVP-Ag–CuO NCS was found most effective in the arrest of cellular proliferation in a dose-dependent manner, amplification of cell hammering viability was experimental with magnification in dose concentration.

### Effect of nanomaterials treatment on lipid peroxidation in HepG2 cancer cells

Lipid peroxidation is triggered by reactive oxygen species (ROS) and plays a crucial role in cell death, including autophagy and apoptosis. This vital and well-preserved process is based on the production of excessive ROS, which causes bio-membrane damage, promotes lipid peroxidation chain events, and ultimately leads to cell death. The results of the present study revealed that AgNPs, CuONPs, and polymeric PVP-Ag–CuO NCS exerted a potent dose-dependent effect on lipid peroxidation in HepG2 cancer cells. Among the three nanomaterials, a noticeable elevation of lipid peroxidation with the rise in a dose of polymeric PVP-Ag–CuO NCS followed by CuONPs and AgNPs treatment (100 and 200 µg mL^−1^) was observed in HepG2 cancer cells (Fig. 9B).

### Depletion of intracellular glutathione (GSH) of HepG2 cancerous cells

GSH redox is an important component in a variety of biological activities, including regulation of cell proliferation and apoptosis, control of numerous signal transduction pathways, and gene initiation at the transcription level. The results of the current study showed that the treatment AgNPs, CuONPs, and polymeric PVP-Ag–CuO NCS potentially depleted the levels of GSH in HepG2 cancer cells, which suggested that all the three pre-synthesized nanomaterials severed as potent anticancer agents against cancer cells. At 200 µg mL^−1^, the statistically potential depletion of 58.37%, 53.77%, and 47.57% in the GSH levels was observed for polymeric PVP-Ag–CuO NCS, AgNPs, and CuONPs treated HepG2 cancer cells, respectively. While at 100 µg mL^−1^, 38.76%, 34.17%, and 20.86% decrease in GSH levels was noticed after the treatment of polymeric PVP-Ag–CuO NCS, AgNPs, and CuONPs in HepG2 cancer cells, respectively (Fig. 9C). Redox state (one of the main components being GSH) is a key factor of metastatic aggressiveness and chemotherapeutic susceptibility. The decrease in GSH/glutathione disulfide (GSSG) ratio or GSH deficit, results in an elevated susceptibility to the oxidative stress related to cancer growth, and increased GSH intensities which amplify the antioxidant ability and resistance to the oxidative stress as pragmatic in many types of cancerous cells. GSH is a well-known oxidative stress marker and an important ROS scavenger. The results obtained in this study support earlier studies by reducing intracellular GSH levels in HepG2 cancer cells after the treatment with polymeric PVP-Ag–CuO NCS, CuONPs, and AgNPs. Thus, posing cells become more vulnerable to ROS production, resulting in apoptosis (Fig. 9D). The overall results give a preliminary confirmation view that cellular GSH expression in cancerous cells may be a target for therapeutic operations (Niu et al., 2021). The outcomes support that AgNPs, CuONPs, and polymeric Ag–CuO NCS may contribute as therapeutic anticancer agents. However, the Ag–CuO NCS exhibit results compared to AgNPs and CuONPs in a dose-dependent manner. The potent cytotoxic effect of polymeric Ag–CuO NCS is the result of combined active physiochemical interaction of silver (Ag^+^) ions, copper oxide (Cu^2+^) ions, bioactive constituents of *L. dendroidea* extract with the functional groups of intracellular proteins, nitrogen bases and phosphate groups in DNA (Jadhav et al., 2018). An earlier study reported by Sriram et al. reported that the nanomaterials possessing anticancer potentials are known for their significant capability to lower the activities of abnormally shown by signaling proteins, including Akt and Ras, DNA- or protein-based vaccines towards specific tumor markers, cytokine-based therapies, and tyrosine kinase inhibitors which display a consistent antitumor activity (Sriram et al., 2010). In this study, the significant anticancer potential was observed and the pre-synthesized, AgNPs, CuONPs, and polymeric Ag–CuO NCS induce a dose dependent inhibition effect against HepG2 liver cancer. Although, some of the approved reported chemotherapeutic agents can cause side effects and are expensive. Therefore, there is an urgent need to develop alternative medicines against this deadly disease. The biosynthesized AgNPs, CuONPs, and polymeric PVP-Ag–CuO NCS could fulfill the need for new therapeutic treatment after clinical exploration. The previous studies have addressed the promising cytotoxicity of green synthesized AgNPs against HepG2 liver cancer cell line with IC50 values in the range of 5–50 µg mL^−1^ (Al-Khedhairy & Wahab, 2022; Ananthi & Iswarya, 2019). Whereas, the green synthesized CuONPs showed moderate anticancer potential with IC50 values in the range of 5–15 µg mL^−1^ against a similar cell line (Abudayyak, Guzel & Ozhan, 2020). However, there is no study reported for Ag–CuONPs polymeric bionanocomposite using *L. dendroidea* extract. Our study showed that all three formed nanomaterials exhibit good anticancer potential with IC50 values of 41.25, 52.14, and 38.12 for AgNPs, CuONPs, and polymeric PVP-Ag–CuO NCS, respectively. The excellent anticancer activity of polymeric PVP-Ag–CuO NCS in contrast to AgNPs and CuONPs, could be attributed to the synergetic effect components of *L. dendroidea* extract with Ag, CuO, and Ag–CuO blend.

### Photocatalytic activity

**Figure 10:**
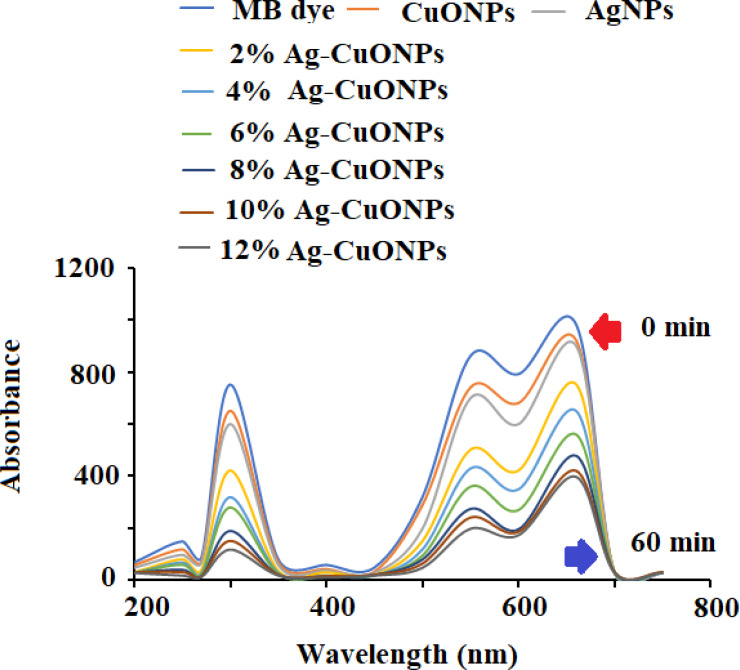
Photocatalytic degradation of MB dye using different concentrations of Ag–CuO bionanocomposite (2%–12%).

The photocatalytic potential of AgNPs, CuONPs, and polymeric Ag–CuO NCS was evaluated by the photocatalytic decomposition of MB under visible light. The results revealed that the photocatalytic effect of AgNPs was closely dependent on irradiation time. As depicted in Fig. 10, the absorption spectra at varied time gaps of an aqueous solution of MB in the presence of undoped and doped CuONPs with varied concentrations of AgNPs (2%–12%) and the characteristic absorption peak (*λ*_max_) for MB at 662 nm was recorded. The rate of degradation of MB dye was very fast in the initial step and then the rate started slowing down until no degradation was noticed after 60 min. As evident from the literature, doping with AgNPs reduces the bandgap of CuONPs. The doping slows down the rapid recombination of photoinduced electron–hole pairs in the produced samples, which enhances the photocatalytic efficiency of the polymeric PVP-Ag–CuO NCS. It is evident from the spectra that Ag–CuO bionanocomposite exhibit better degradation performance in comparison to CuONPs and AgNPs. The availability of photo-induced charge carriers for the photocatalytic degradation of MB dye may also be improved by the greater BET surface area of Ag–CuONPs (24.20 m^2^ g^−1^) compared to AgNPs (14.68 m^2^g^−1^) and CuONPs (12.80 m^2^ g^−1^) (Liu et al., 2020; Meng et al., 2020; Li, Chen & Li, 2019). The photoluminescence (PL) spectra demonstrate that the Ag (4%) concentration is sufficient for the efficient separation and transportation of electron–hole pairs. The obtained results showed that the doping of Ag increases the p-type conductivity and 4% is the optimum concentration for doping. Thus, increase in Ag^+^ ion concentration of more than 4% resulted in the decrease photocatalytic effect and impact the production of less active polymeric Ag–CuO NCS photocatalyst. The incorporation of Ag into the host CuO lattice causes stress in the crystal structure due to the greater ionic radius of Ag^+^ than Cu^2+^. After 60 min irradiation, the maximum rate of MB degradation was 65% over polymeric PVP-Ag–CuO NCS (Fig. 10).

## Conclusion

The green ecofriendly biogenic synthesis of AgNPs, CuONPs, and polymeric PVP-Ag–CuO NCS was performed by using macroalga *L. dendroidea*. The formed nanomaterials were characterized and confirmed by different analytical procedures such as UV-vis, FTIR, XRD, zeta potential, SEM combined with EXD and TEM. The pre-synthesized AgNPs, CuONPs, and polymeric PVP-Ag–CuO NCS were found to be spherical in shape. The SEM and TEM images of formed nanomaterials were spherical in shape with a little agglomeration. The EDX spectrum of polymeric PVP-Ag–CuO NCS strongly represents silver and copper oxide metals present in the polymeric PVP-Ag–CuO NCS matrix. Furthermore, antiprotozoal and anticancer evaluation of nanomaterials revealed the polymeric PVP-Ag–CuO NCS expressed excellent antiprotozoal and anticancer activities when compared to AgNPs and CuONPs The polymeric PVP-Ag–CuO NCS exhibited strong antiprotozoal effect against both the *T.cruzi* and *L. amazonensis* parasites with IC50 values of 17.32 ± 1.0 and 17.48 ± 0.9 µg mL^−1^, at 4.2 and 3.9 µg mL^−1^, respectively. These nanomaterials (AgNPs, CuONPs, and polymeric PVP-Ag–CuO NCS) were found to be effective towards HepG2 cancerous cells as well as protozoans. However, the highest anticancer activity was exerted by PVP-Ag–CuO NCS with IC50 values 38.12 ± 0.13 µg mL^−1^ at 200 µg mL^−1^ concentrations. Additionally, the nanomaterials (AgNPs, CuONPs, and polymeric PVP-Ag–CuO NCS) exhibited promising photocatalytic properties with high effect displayed by bionanocomposite. Thus, the outcomes of this study suggested that the pre-synthesized nanomaterials can be further explored for various biomedical applications.

##  Supplemental Information

10.7717/peerj.15004/supp-1 File S1 Raw data for anti-protozoal activity of AgNPs, CuONPs and PVP-Ag/CuONPs nanocomposite against *T. cruzi* epimastigotes

10.7717/peerj.15004/supp-2 File S2 Raw data for anti-protozoal activity of AgNPs, CuONPs and PVP-Ag/CuONPs nanocomposite against *L. amazonensis* promastigotes

10.7717/peerj.15004/supp-3 File S3 Cell viability % of AgNPs, CuONPs, and Ag-CuO nanocomposite against HepG2 cancer cell line at different concentrations (50–200 µg mL^−1^)

10.7717/peerj.15004/supp-4 File S4 Effect on lipid peroxidation in AgNPs, CuONPs, and Ag-CuO nanocomposite treated HepG2 cells at different concentrations

10.7717/peerj.15004/supp-5 Figure S1 DLS spectra (a) AgNPS (b) CuONPs (c) Ag-CuO NCS

10.7717/peerj.15004/supp-6 File S5 Percent change in glutathione depletion in AgNPs, CuONPs, and Ag-CuO nanocomposite treated HepG2 cells at different concentrations

## Additional Information and Declarations

### Data Availability

The following information was supplied regarding data availability: The raw data are available in the Supplemental Files.
