# Supplementary material for: Advanced polymeric metal/metal oxide bionanocomposite using seaweed Laurencia dendroidea extract for antiprotozoal, anticancer, and photocatalytic applications
Source: PeerJ. 2023 Mar 20;11:e15004. doi: 10.7717/peerj.15004 (PMC10035428; doi:10.7717/peerj.15004)

			Size (d.nm):	% Intensity:	St Dev (d.nm):
Z-Average (d.nm): 25.92		Peak 1:	25.92	100.0	481.6
PdI:	0.090	Peak 2:	0.000	0.0	0.000
Intercept:	0.918	Peak 3:	0.000	0.0	0.000
Result quality :	Good				

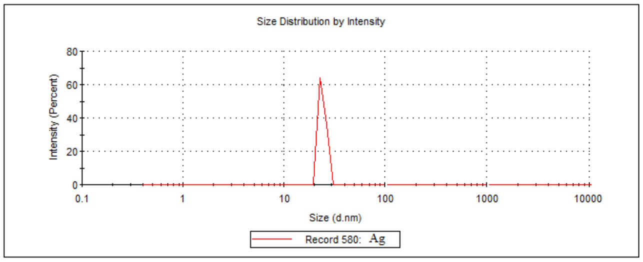

			Size (d.nm):	% Intensity:	St Dev (d.nm):
Z-Average (d.nm):	32.84	Peak 1:	32.15	100.0	45.26
PdI:	0.149	Peak 2:	0.000	0.0	0.000
Intercept:	0.663	Peak 3:	0.000	0.0	0.000
Result quality :	Good				

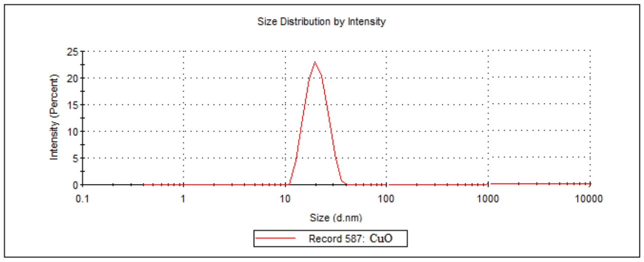

			Size (d.nm):	% Intensity:	St Dev (d.nm):
Z-Average (d.nm):	35.20	Peak 1:	35.20	100.0	45.06
PdI:	0.061	Peak 2:	0.000	0.0	0.000
Intercept:	0.925	Peak 3:	0.000	0.0	0.000
Result quality :	Good				

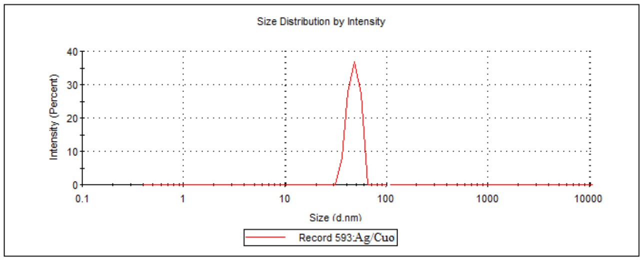

Supplement: Figure S1 — (a) AgNPS (b) CuONPs (c) Ag-CuO NCS [file peerj-11-15004-s005.pdf]
